# Optimizing training sets for genomic selection to identify superior genotypes across multiple environments

**DOI:** 10.1093/g3journal/jkag031

**Published:** 2026-02-10

**Authors:** Zi-Jie Liu, Chen-Tuo Liao

**Affiliations:** Department of Agronomy, National Taiwan University, Taipei 106319, Taiwan; Department of Agronomy, National Taiwan University, Taipei 106319, Taiwan

**Keywords:** genomic prediction, genotype-by-environment interaction, multienvironment trial, plant breeding, training set optimization

## Abstract

Genomic selection (GS) is a promising strategy in plant breeding for identifying superior genotypes with high true breeding values (TBVs) across multiple environments. However, the relative performance of candidate genotypes often varies due to complex genotype-by-environment (G × E) interactions in multienvironment trials (METs). To address this challenge, we employed a GS prediction model incorporating fixed environment-specific means, random additive genetic effects, and random additive G × E interaction effects to develop training set optimization methods for GS in METs. Two optimization methods derived from the generalized coefficient of determination (CD) criterion—$CD_{\mathrm{mean}(v2)}$ (Chen et al. 2024, equivalent to Rincent et al. 2012) and $CD_{\mathrm{mean}.\mathrm{MET}}$ (Rio et al. 2022)—were evaluated and compared with random sampling. Rather than relying on prediction accuracy–focused correlation metrics, we assessed training set performance using selection-focused ranking metrics, including normalized discounted cumulative gain, Spearman's rank correlation, and rank sum ratio. Because TBVs are latent and unobservable, simulation experiments were conducted using real genotype data from diverse crop datasets, including rice (*Oryza sativa* L.), barley (*Hordeum vulgare* L.), and maize (*Zea mays* L.). Among the evaluated approaches, $CD_{\mathrm{mean}(v2)}$ consistently showed high efficiency in identifying top-performing genotypes. In practice, $CD_{\mathrm{mean}(v2)}$, implemented using the optimization algorithm provided in the TrainSel package (Akdemir et al. 2021), is recommended for GS-assisted breeding programs, as it produced superior training sets for identifying elite genotypes with reasonable computational cost.

## Teaser

The selection of superior genotypes that perform well across diverse environments is a critical goal in plant breeding. Genomic selection (GS) has emerged as an innovative and promising strategy to achieve this objective. In this study, Liu and Liao propose a cost-effective and optimal approach for determining training sets to enhance the application of GS in METs. The proposed approach could help plant breeders to select suitable genotypes for selective phenotyping, and then increase the success probability of a GS program applied to multi-environment trials.

## Introduction

Genotype-by-environment (G × E) interaction plays a critical role in plant breeding, as genotypes often display different relative performances across environments. When G × E interaction is weak, breeders tend to favor genotypes with consistently superior performance across environments. In contrast, strong G × E interaction may lead to the selection of genotypes adapted to specific environments. To quantify and characterize such interactions, multienvironment trials (METs) are routinely conducted. A range of statistical approaches has been developed for analyzing MET data. Classical linear models that incorporate genotype and environment main effects, together with bilinear terms for G × E interaction effects, include the Finlay–Wilkinson model (Finlay and Wilkinson 1963), the additive main effects and multiplicative interaction (AMMI) model (Gauch 1988, 2006), and the genotype main effect plus G × E interaction (GGE) model (Yan 2001; Yan et al. 2007). In addition to treating these effects as fixed, they can also be modeled as random effects within a linear mixed model framework. Linear mixed models further allow the incorporation of heterogeneous genetic variances, kinship among genotypes, correlations between environments, block effects in experimental designs, and spatial variation within fields (Butler et al. 2014; Cullis et al. 2020). Consequently, linear mixed models have become a widely applied and powerful framework for G × E analysis (van Eeuwijk et al. 2016).

Genomic selection (GS) provides an efficient strategy to predict breeding values across a wide range of G × E scenarios and is therefore highly valuable for selecting genotypes with superior and stable performance (Burgueno et al. 2008; Heslot et al. 2013; Malosetti et al. 2016). The central concept of GS is to capture the effects of quantitative trait loci using dense molecular markers across the genome (Meuwissen et al. 2001). Genomic estimated breeding values (GEBVs) are predicted by fitting models to phenotypic and genotypic data from a training set. The accuracy of these predictions strongly depends on the size and representativeness of the training set. Because phenotyping in field trials is often constrained by resources, optimizing training sets to achieve cost-effective selective phenotyping is essential for improving the efficiency of GS (Wu et al. 2023).

Previous research has extensively investigated training set optimization strategies aimed at maximizing the correlation between true breeding values (TBVs) and GEBVs, a key determinant of genetic gain (Heffner et al. 2010). Under the linear mixed model framework—commonly referred to as genomic best linear unbiased prediction (GBLUP) in GS—Rincent et al. (2012), Isidro et al. (2015), and Rincent et al. (2017) proposed optimization methods based on the generalized coefficient of determination (CD) (Laloë 1993). Alternatively, within whole-genome regression (WGR) models that express genotypic values as linear combinations of fixed marker effects, Akdemir et al. (2015) and Ou and Liao (2019) developed training set optimization strategies tailored to GS. Other approaches have focused on maximizing genomic relationships between training and testing sets (Atanda et al. 2021) or on Bayesian optimization methods (Tanaka and Iwata 2018; Tsai et al. 2021; Tu and Liao 2024). A comprehensive comparison of these approaches was provided by Fernández-González et al. (2023). However, most existing methods are restricted to single-environment models that consider only marker main effects.

Akdemir et al. (2021) developed the TrainSel package, which provides a flexible and efficient software framework for implementing training set optimization methods. The default CD criterion implemented in TrainSel is $CD_{\min}$, defined as the minimum squared correlation between the true and estimated genotypic values among the selected genotypes. Maximizing $CD_{\min}$ follows a maximin strategy, improving the worst-case prediction accuracy and supporting risk-averse decision-making. In addition to $CD_{\min}$, TrainSel offers alternative optimization criteria, including $CD_{\mathrm{mean}}$ (Rincent et al. 2012) and the r-score (Ou and Liao 2019). Both $CD_{\min}$ and $CD_{\mathrm{mean}}$can be directly applied to selective phenotyping and MET settings with environment-specific and unbalanced allocations. Rio et al. (2022) further proposed several extensions of the CD criterion for MET optimization and demonstrated their flexibility in accommodating environment-specific and variable allocation sizes. Nevertheless, most prior studies have evaluated training set performance primarily using prediction accuracy–focused correlation metrics for targeted or untargeted optimization methods. A comprehensive assessment of training set optimization in METs using selection-focused ranking metrics across diverse crop datasets remains limited.

Several extensions have sought to incorporate G × E effects into GS prediction models. López-Cruz et al. (2015) proposed a WGR model that included marker-by-environment interaction terms, assuming homogeneous residual variances across environments. Crossa et al. (2016) relaxed this assumption by allowing heterogeneous residual variances, while Aleves et al. (2021) applied GBLUP models aligned with these WGR formulations to evaluate additive and dominance marker effects in MET data. These models, which account for environment-specific variation in marker effects, are often referred to as marker G × E (MGE) models. More recently, Rio et al. (2022) compared several GBLUP models for G × E analysis and examined training set optimization in METs using CD-based criteria. Building on this foundation, the present study evaluates 2 CD-based optimization methods proposed by Rio et al. (2022) for GS in METs within the MGE model framework. We further assess the ability of the resulting training sets to identify superior genotypes with high TBVs through simulation experiments based on real genotype data. Training set performance is evaluated using 3 selection-focused ranking metrics—normalized discounted cumulative gain (NDCG), Spearman's rank correlation (SRC), and rank sum ratio (RS_ratio_)—across 3 crop datasets: rice (*Oryza sativa* L.), barley (*Hordeum vulgare* L.), and maize (*Zea mays* L.).

## Materials and methods

Genotype data from 3 publicly available crop datasets were used as templates for the simulation experiments, as these datasets represent relatively homogeneous genomes and are therefore well suited for fitting the additive marker effects model considered in this study.

### Tropical rice

Chung and Liao (2020) analyzed a tropical rice dataset for identifying superior parental lines, originally published by Spindel et al. (2015). The dataset contained 73,147 single nucleotide polymorphism (SNP) markers and 363 elite breeding lines belonging to the *indica* or *indica*-admixed group. Phenotypic observations were conducted 8 times in 2009 to 2012, once in the dry season and once in the wet season each year, on grain yield, flowering time, and plant height (PH), although PH data were not available for the wet season of 2009. Phenotypic values for 35 of the 363 individuals were missing. To reduce marker density, Chung and Liao (2020) randomly selected 1 SNP per 0.1-cM interval across each chromosome, yielding 10,772 SNPs. For our simulation study, we used the filtered dataset consisting of 328 genotypes and 10,772 SNPs.

### Barley

Oakey et al. (2016) used a barley PH dataset to demonstrate GS-based MET analysis. The dataset included 477 spring barley lines evaluated for PH across 2 consecutive years (2010 and 2011). Of these, 456 lines with phenotypic records in both years were retained for analysis. The original genotypic dataset contained 3,490 high-confidence gene-based SNP markers. After excluding markers with minor allele frequency (MAF) < 5%, 3,469 SNPs remained and were used in our simulation study.

### DST2 maize

Rio et al. (2022) employed a maize dataset, originally presented by Jarquin et al. (2020), to evaluate prediction accuracy of different GBLUP models in METs. The dataset, referred to as DST2, comprised 453 CIMMYT hybrids derived from crossing tester genotype T2 with 453 other lines. Grain yield was measured across 3 environments. A total of 73,919 SNP markers were available, and due to the high genetic homogeneity of the dataset, only additive marker effects were considered in Rio et al. (2022). After filtering SNPs with MAF < 5%, 62,882 markers were retained for our simulation study.

### Training set optimization methods

After preprocessing steps, including quality filtering, imputation, and normalization of the original genotype data, SNPs at each locus were recoded as −1, 0, and 1, corresponding to the homozygote for the minor allele, the heterozygote, and the homozygote for the major allele, respectively. Let $X_{cp}$ be the original marker score matrix and $W_{cp}$ be the standardized marker score matrix. That is, $w_{ij}=\frac{x_{ij}-{\bar{x}}_{j}}{s_{j}}$, where $w_{ij}$ and $x_{ij}$ are respectively the (ij)th elements in $W_{cp}$ and $X_{cp}$ for $i=1,\;2,\;\ldots ,\;n_{cp}$ and j=1,2,…,p. Here, ${\bar{x}}_{j}$ and $s_{j}$ are respectively the sample mean and the sample standard deviation for column *j* in $X_{cp}$, $n_{cp}$ is the number of genotypes in the genotype dataset, and *p* is the number of markers. The genomic relationship matrix of the candidate population was then calculated by $K_{cp}=\frac{1}{p}(W_{cp}W_{cp}^{T})$.

Rio et al. (2022) employed the $CD_{\mathrm{mean}}$ criterion for training set optimization in METs, which was originally proposed by Rincent et al. (2012) for single-environment settings. More recently, Chen et al. (2024) introduced the $CD_{\mathrm{mean}(v2)}$ criterion for single-environment optimization and provided a formal mathematical proof of its equivalence to $CD_{\mathrm{mean}}$, together with a detailed analysis of computational complexity. In this study, we adopt $CD_{\mathrm{mean}(v2)}$ for MET optimization within the MGE model framework. Let $S_{tr}$ denote a training set consisting of $S_{1},\;S_{2}\;\cdots ,$ and $S_{T},\;$ where $S_{j}$ is the subset of $S_{tr}$ in environment *j* for j=1,2,⋯,T. Here, *T* is the number of environments. The total training set size $n_{tr}=n_{1}+n_{2}+\cdots +n_{T},$ where $n_{j}$ is the size of $S_{j}$. The MGE model for fitting the training set can be described as follows:


(1) $$y_{tr}=\mu_{tr}+a_{tr}+b_{tr}+e_{tr},$$

where $y_{tr}=\left[\begin{array}{c} y_{1}\\ \vdots \\ y_{T} \end{array}\right]$ denotes the phenotypic values in $S_{tr}$, and $y_{j}$ is the subvector of $y_{tr}$ representing the phenotypic values in $S_{j}$; $\mu_{tr}=\left[\begin{array}{c} \mu_{1}1_{n_{1}}\\ \vdots \\ \mu_{T}1_{n_{T}} \end{array}\right]$ denotes the fixed environmental means, $\mu_{j}$ represents the true mean of phenotypic values in environment *j*, and $1_{n_{j}}$ is the unit vector of length $n_{j}$; $a_{tr}$ is the vector of genotypic values associated with the additive effects; $b_{tr}$ is the vector of genotypic values associated with the additive G × E effects; and $e_{tr}$ is the vector of environmental residuals. It is assumed that $a_{tr}$, $b_{tr}$, and $e_{tr}$ are mutually independent and follow multivariate normal distributions which can be described as follows:

(i) $a_{tr}∼MVN(0,\;V_{a})$ with
  $$V_{a}=\left[\begin{array}{cccc} \;\sigma_{0}^{2}K_{1} & 0 & \cdots & 0\\ 0 & \;\sigma_{0}^{2}K_{2} & \cdots & 0\\ \vdots & \vdots & \ddots & \vdots \\ 0 & 0 & \cdots & \;\sigma_{0}^{2}K_{T} \end{array}\right],$$

where $\sigma_{0}^{2}$ is the homogeneous genetic variance for the additive effects, and $K_{j}$ is the genomic relationship matrix for $S_{j}$ for j=1,2,⋯,T.

(ii) $b_{tr}∼MVN(0,\;V_{b})$ with
  $$V_{b}=\left[\begin{array}{cccc} \sigma_{1}^{2}K_{1} & \tau_{0}K_{12} & \cdots & \tau_{0}K_{1T}\\ \tau_{0}K_{21} & \sigma_{2}^{2}K_{2} & \cdots & \tau_{0}K_{2T}\\ \vdots & \vdots & \ddots & \vdots \\ \tau_{0}K_{T1} & \tau_{0}K_{T2} & \cdots & \sigma_{T}^{2}K_{T} \end{array}\right],$$

where $\sigma_{j}^{2}$ is the genetic variance in environment *j* for the additive G × E effects, $\tau_{0}$ is the common genetic covariance between 2 environments for the additive G × E effects, and $K_{\;j\overset{´}{j}}$ is the genomic relationship matrix between $S_{j}$ and $S_{\overset{´}{j}}$ for $1\leq j\neq \overset{´}{j}\leq \;T$.

(iii) $e_{tr}∼MVN(0,\;R_{E})$ with
  $$R_{E\;}=\left[\begin{array}{cccc} \sigma_{E1}^{2}I_{n_{1}} & 0 & \cdots & 0\\ 0 & \sigma_{E2}^{2}I_{n_{2}} & \cdots & 0\\ \vdots & \vdots & \ddots & \vdots \\ 0 & 0 & \cdots & \sigma_{ET}^{2}I_{n_{T}} \end{array}\right],$$

where $\sigma_{Ej}^{2}$ is the variance of residuals in environment *j*, and $\;I_{n_{j}}$ is the identity matrix of order $n_{j}$ for j=1,2,⋯,T.

Let $g_{tr}=a_{tr}+b_{tr},$ and $\sigma_{G\times j}^{2}=\sigma_{0}^{2}+\sigma_{j}^{2}$ for j=1,2,⋯,T, and then the MGE model in Eq. (1) can be equivalently written as:


(2) $$y_{tr}=\mu_{tr}+g_{tr}+e_{tr},$$

where $g_{tr}$ consists of the pooled genotypic values in $S_{tr},$ and $g_{tr}∼MVN(0,\;G_{tr})$ with


$$G_{tr}=\left[\begin{array}{cccc} \sigma_{G\times 1}^{2}K_{1} & \tau_{0}K_{12} & \cdots & \tau_{0}K_{1T}\\ \tau_{0}K_{21} & \sigma_{G\times 2}^{2}K_{2} & \cdots & \tau_{0}K_{2T}\\ \vdots & \vdots & \ddots & \vdots \\ \tau_{0}K_{T1} & \tau_{0}K_{T2} & \cdots & \sigma_{G\times T}^{2}K_{T} \end{array}\right].$$

From Henderson's mixed model equations (Henderson 1975), the BLUP for $g_{tr}$ is given by:


$${\hat{g}}_{tr}=(M_{tr}+G_{tr}^{-1}{)}^{-1}M_{tr}y_{tr},$$

where


$$M_{tr}=\left[\begin{array}{cccc} {\left(\sigma_{E1}^{2}\right)}^{-1}(I_{n_{1}}-{\bar{J}}_{n_{1}}) & 0 & \cdots & 0\\ 0 & {\left(\sigma_{E2}^{2}\right)}^{-1}(I_{n_{2}}-{\bar{J}}_{n_{2}}) & \cdots & 0\\ \vdots & \vdots & \ddots & \vdots \\ 0 & 0 & \cdots & {\left(\sigma_{ET}^{2}\right)}^{-1}(I_{n_{T}}-{\bar{J}}_{n_{T}}) \end{array}\right].$$

Here, $M_{tr}$ is a block diagonal matrix with the $j^{th}$ diagonal submatrix $\left(\sigma_{Ej}^{2}{)}^{-1}(I_{n_{j}}-{\bar{J}}_{n_{j}}\right)$ orthogonal to the corresponding $j^{th}$ subvector $\mu_{j}1_{n_{j}}$ in $\mu_{tr},\;$ and ${\bar{J}}_{n_{j}}$ is the square matrix of order $n_{j}$ with all elements equal to $1/n_{j}$. Let $h_{j}$ denote the true genotypic values of the candidate population in environment *j* for j=1,2,⋯,T. Moreover, let $h_{cp}$ consist of $h_{1},\;h_{2},\;\cdots ,$ and $h_{T},$ and then $h_{cp}$ follows a multivariate normal distribution, denoted by $h_{cp}∼MVN(0,\;G_{cp})$ with


$$G_{cp}=\left[\begin{array}{cccc} \sigma_{G\times 1}^{2}K_{cp} & \tau_{0}K_{cp} & \cdots & \tau_{0}K_{cp}\\ \tau_{0}K_{cp} & \sigma_{G\times 2}^{2}K_{cp} & \cdots & \tau_{0}K_{cp}\\ \vdots & \vdots & \ddots & \vdots \\ \tau_{0}K_{cp} & \tau_{0}K_{cp} & \cdots & \sigma_{G\times T}^{2}K_{cp} \end{array}\right]$$

(3) $$=\Omega_{G}⊗K_{cp},$$

where ⊗ denotes the Kronecker product,


$$\Omega_{G}=\;\left[\begin{array}{cccc} \sigma_{G\times 1}^{2} & \tau_{0} & \cdots & \tau_{0}\\ \tau_{0} & \sigma_{G\times 2}^{2} & \cdots & \tau_{0}\\ \vdots & \vdots & \ddots & \vdots \\ \tau_{0} & \tau_{0} & \cdots & \sigma_{G\times T}^{2} \end{array}\right],$$

and $K_{cp}$ is the genomic relationship matrix for the candidate population. From Henderson (1977), the BLUP for $h_{cp}$ is obtained as:


$${\hat{h}}_{cp}=G_{cp.tr}G_{tr}^{-1}{\hat{g}}_{tr}=G_{cp.tr}(M_{tr}G_{tr}+I_{n_{tr}}{)}^{-1}M_{tr}y_{tr},$$

where $G_{cp.tr}$ is the covariance matrix between $h_{cp}$ and $g_{tr}$, which can be expressed as:


. $$G_{cp.tr}=\;\left[\begin{array}{cccc} \sigma_{G\times 1}^{2}K_{cp.1} & \tau_{0}K_{cp.2} & \cdots & \tau_{0}K_{cp.T}\\ \tau_{0}K_{cp.1} & \sigma_{G\times 2}^{2}K_{cp.2} & \cdots & \tau_{0}K_{cp.T}\\ \vdots & \vdots & \ddots & \vdots \\ \tau_{0}K_{cp.1} & \tau_{0}K_{cp.2} & \cdots & \sigma_{G\times T}^{2}K_{cp.T} \end{array}\right]$$

Here, $K_{cp.j}$ is the genomic relationship matrix between the candidate population and $S_{j}$ for j=1,2,⋯,T. It can be verified that the variance–covariance matrix for ${\hat{h}}_{cp}$ is the same as the covariance matrix between $h_{cp}$ and ${\hat{h}}_{cp}$, which is given by:


$$Var({\hat{h}}_{cp})=Cov(h_{cp},{\hat{h}}_{cp})$$

(4) $$=\;G_{cp.tr}(M_{tr}G_{tr}+I_{n_{tr}}{)}^{-1}M_{tr}G_{cp.tr}^{T}$$

Furthermore, let $A_{l,l^{\prime }}$ denote the element at row l and column $l^{\prime }$ in the matrix of Eq. (4), and $B_{l,l^{\prime }}$ denote the corresponding element in $G_{cp}$ of Eq. (3), for $l,l^{\prime }=1,\;\cdots ,n_{cp}+1,\cdots ,(T-1)\times n_{cp}+1,\cdots ,\;N,$ where $\;N=T\times n_{cp}$. Let $h_{ij}$ and ${\hat{h}}_{ij}$ denote the true and estimated genotypic values of a genotype *i* in environment *j* for $i=1,\;\cdots ,n_{cp}$, and j=1,⋯,T. From Chen et al. (2024), an optimization criterion for maximizing the mean squared correlation between the true and estimated genotypic values over genotypes and environments is given by:


$$CD_{\mathrm{mean}(v2)}=\overset{n_{cp}}{\underset{i=1}{\sum }}\;\overset{T}{\underset{\;j=1}{\sum }}\frac{{\left[cov(h_{ij},{\hat{h}}_{ij})\right]}^{2}}{var(h_{ij})\times var({\hat{h}}_{ij})}$$

$$=\overset{N}{\underset{l=1}{\sum }}\;(\frac{{\left[A_{l,l}\right]}^{2}}{B_{l,l}\times A_{l,l}})$$

(5) $$=\overset{N}{\underset{l=1}{\sum }}\;(\frac{A_{l,l}}{B_{l,l}}).$$

Rio et al. (2022) also proposed an alternative form of CD for optimizing experimental designs in METs. This criterion was defined as the mean squared correlation between the environment-averaged true and estimated genotypic values. Here, we refer to this criterion as $CD_{\mathrm{mean}.\mathrm{MET}}$, which can be expressed as:


$$CD_{\mathrm{mean}.\mathrm{MET}}=\overset{n_{c}}{\underset{i=1}{\sum }}\frac{{\left[cov({\bar{h}}_{i.},{\bar{\hat{h}}}_{i.})\right]}^{2}}{var({\bar{h}}_{i.})\times var({\bar{\hat{h}}}_{i.})}$$

$$=\overset{n_{c}}{\underset{i=1}{\sum }}\;\left(\frac{{\left[A_{i}^{*}\right]}^{2}}{B_{i}^{*}\times A_{i}^{*}}\right)$$

(6) $$=\overset{n_{c}}{\underset{i=1}{\sum }}\;(\frac{A_{i}^{*}}{B_{i}^{*}}),$$

where ${\bar{h}}_{i.}=\frac{1}{T}\overset{T}{\underset{\;j=1}{\sum }}\;h_{ij},$ and ${\bar{\hat{h}}}_{i.}=\frac{1}{T}\overset{T}{\underset{\;j=1}{\sum }}\;{\hat{h}}_{ij}$ are respectively the averages of the true and estimated genotypic values for genotype *i* over the *T* environments,


$$A_{i}^{*}=\overset{T}{\underset{\;j=1}{\sum }}\;\overset{T}{\underset{m=1}{\sum }}\;A_{(\;j-1)\times n_{c}+i,\;(m-1)\times n_{c}+i}$$

and


$$B_{i}^{*}=\overset{T}{\underset{\;j=1}{\sum }}\;\overset{T}{\underset{m=1}{\sum }}\;B_{(\;j-1)\times n_{c}+i,\;(m-1)\times n_{c}+i}.$$

The variance components $\sigma_{G\times j}^{2}$ and $\sigma_{Ej}^{2}$ were fixed at 1 for j=1,2,⋯,T, and the common genetic covariance $\tau_{0}$ was fixed at 0.5 when calculating $CD_{\mathrm{mean}(v2)}$ in Eq. (5) and $CD_{\mathrm{mean}.\mathrm{MET}}$ in Eq. (6). This choice was motivated by previous findings showing that training set selection based on CD-based criteria is relatively insensitive to the specification of these parameter values (Rincent et al. 2012; Rio et al. 2022; Fernández-González et al. 2023). The robustness of this setting is further discussed in the final section. Identifying globally optimal training sets under $CD_{\mathrm{mean}(v2)}$ and $CD_{\mathrm{mean}.\mathrm{MET}}$ is computationally infeasible using exhaustive search algorithms. Therefore, we employed a metaheuristic search approach based on the genetic algorithm (GA) proposed by Ou (2022) to obtain locally optimal training sets. This GA-based exchange algorithm has been shown to be effective for implementing heuristic training set optimization methods (Ou and Liao 2019; Wu et al. 2023; Chen et al. 2024); however, it can incur substantial computational cost when applied to large datasets. Notably, $CD_{\mathrm{mean}(v2)}$ and $CD_{\mathrm{mean}.\mathrm{MET}}$ are untargeted optimization methods, as they do not incorporate information from a specific target testing set.

### Evaluation metrics

Three ranking-based metrics were used to assess the performance of a training set in identifying the best *k* genotypes from a candidate population, which have the highest TBVs in a specific environment or mean TBVs across environments. In this study, the best *k* genotypes were designated to include the top 5% genotypes of the candidate population, i.e. *k* is the largest integer less than or equal to $0.05\times n_{cp}$. Let $v_{\left(1\right)}\geq v_{\left(2\right)}\geq \ldots \geq v_{\left(n_{cp}\right)}$ be TBVs of all candidates arranged in descending order. Notably, the TBVs here can be individual values in a specific environment or mean values over environments. The TBVs are unobservable but can be estimated using GEBVs. Furthermore, let ${\hat{v}}_{\left(1\right)}$, ${\hat{v}}_{\left(2\right)}$, …, ${\hat{v}}_{\left(n_{cp}\right)}$ be their corresponding GEBVs obtained using the training dataset. By reordering these GEBVs, it follows that ${\hat{v}}_{\left(\pi_{1}\right)}\geq {\hat{v}}_{\left(\pi_{2}\right)}\ldots \geq {\hat{v}}_{\left(\pi_{n_{cp}}\right)},$ where $\pi =(\pi_{1},\pi_{2},\ldots ,\pi_{n_{cp}})$ is a permutation of $\pi_{0}=(1,\;2,\ldots ,\;n_{cp}).$ Notice that $\pi_{1},\;\pi_{2}\ldots ,\;\;\pi_{n_{cp}}$ are exactly the ranks of TBVs corresponding to ${\hat{v}}_{\left(\pi_{1}\right)},\;{\hat{v}}_{\left(\pi_{2}\right)},\;\ldots ,\;{\hat{v}}_{\left(\pi_{n_{cp}}\right)}.$

### Normalized discounted cumulative gain

From Blondel et al. (2015), the discounted cumulative gain (DCG) score at position *k* of the predicted ranking using the training set is defined as follows:


(7) $$DCG@k(v,\pi (\hat{v}))=\overset{k}{\underset{i=1}{\sum }}\;f(v_{\left(\pi_{i}\right)})d(i),$$

and the DCG score at position *k* of the ideal ranking is given as:


(8) $$DCG@k(v,\pi_{0}(v))=\overset{k}{\underset{i=1}{\sum }}\;f(v_{\left(i\right)})d(i),$$

where f(v) is a monotonically increasing gain function, and d(i) is a monotonically decreasing discount function. The linear gain function of f(v)=v and the discount function of $d(i)=\frac{1}{\mathrm{lo}g_{2}(i+1)}$ are used in Eqs. (7) and (8). Finally, the NDCG score at position *k* is defined as follows:


(9) $$NDCG@k(v,\hat{v})=\frac{DCG@k(v,\pi (\hat{v}))}{DCG@k(v,\pi_{0}(v))}$$

The $NDCG@k(v,\hat{v})$ falls between 0 and 1, a training set with improved performance as its resulting NDCG value increases.

### Spearman's rank correlation

The SRC was applied to measure the linear relationship between the ranks of the top *k* genotypes with the highest GEBVs and their true ranks in TBVs, which was obtained as follows:


$$SRC@k=\frac{\sum_{i=1}^{k}\;\left(i-\frac{k+1}{2}\right)(\pi_{i}-\bar{\pi })}{\sqrt{\left[\sum_{i=1}^{k}\;{\left(i-\frac{k+1}{2}\right)}^{2}]\times [\sum_{i=1}^{k}\;{\left(\pi_{i}-\bar{\pi }\right)}^{2}\right]}},$$

where $\bar{\pi }=\overset{k}{\underset{i=1}{\sum }}\;\pi_{i}/k.$ The SRC@k is actually Pearson's correlation calculated from the paired values of $\left(i,\;\pi_{i}\right)$ for i=1,2,⋯,k.

### Rank sum ratio

The sum of ranks in TBVs corresponding to the top *k* genotypes with the highest GEBVs is equal to $\overset{k}{\underset{i=1}{\sum }}\;\pi_{i}$, and the sum of ranks of the ideal ranking is equal to $\overset{k}{\underset{i=1}{\sum }}\;i$. The ratio of the 2 rank sums indicates the ability of the training set to identify the best *k* genotypes, which is defined as:


$$RS_{ratio}@k=\frac{\sum_{i=1}^{k}\;i}{\sum_{i=1}^{k}\;\pi_{i}}.$$

The $RS_{ratio}@k$ also ranges from 0 to 1, equal to 1 if the *k* genotypes with the highest GEBVs exactly include those top *k* genotypes with the highest TBVs.

### Simulation experiments

The simulation experiments were primarily based on the MGE model described in Eq. (2). Genotypic main effects, environmental main effects, and G × E interaction effects were all included to generate phenotypic data. Effects associated with experimental design such as block effects, or with field heterogeneity such as spatial variation, were not considered. This is because phenotypic values used for GEBV prediction are typically adjusted means, for which block and spatial effects are assumed to have been removed during preprocessing. The TBVs of candidate genotypes are latent quantities derived from the MGE model. Specifically, TBVs represent the expected phenotypic performance of genotypes across environments, combining genotypic main effects and G × E interaction effects.

The following simulation studies were therefore conducted to evaluate the performance of construction methods in identifying superior genotypes with the highest TBVs. Each of the 3 genotype datasets was used as a template to simulate various scenarios in METs. The numbers of environment in this simulation study were fixed at T=2,2,and3 and 3 for the tropical rice, barley, and DST2 maize datasets, respectively. The $CD_{\mathrm{mean}(v2)},\;CD_{\mathrm{mean}.\mathrm{MET}},$ and random sampling methods were employed to construct training sets according to 6 combinations of training set sizes in different environments for the datasets, as described in Table 1.

**Table 1.** The combinations of training set sizes in different trials for the datasets in the simulation experiments.

Combinations	Tropical rice (barley)	DST2 maize
I	$\left(n_{1}=50,\;n_{2}=50\right)$	$\left(n_{1}=50,\;n_{2}=50,n_{3}=50\right)$
II	$\left(n_{1}=50,\;n_{2}=75\right)$	$\left(n_{1}=50,\;n_{2}=50,n_{3}=75\right)$
III	$\left(n_{1}=50,\;n_{2}=100\right)$	$\left(n_{1}=50,\;n_{2}=50,n_{3}=100\right)$
IV	$\left(n_{1}=75,\;n_{2}=100\right)$	$\left(n_{1}=50,\;n_{2}=75,n_{3}=100\right)$
V	$\left(n_{1}=100,\;n_{2}=100\right)$	$\left(n_{1}=50,\;n_{2}=100,n_{3}=100\right)$
VI	$\left(n_{1}=100,\;n_{2}=150\right)$	$\left(n_{1}=100,\;n_{2}=100,n_{3}=100\right)$

Based on the MGE model in Eq. (2), TBVs and phenotypic values were simulated. For a given candidate population, the genomic relationship matrix $K_{cp}$ was first calculated. The model parameters were fixed at $\mu_{1}=100,\;\mu_{2}=150,\;\tau_{0}=10,\;\sigma_{G\times 1}^{2}=20,\;\sigma_{G\times 2}^{2}=10,\;20,\;30,$ and $\sigma_{Ej}^{2}=\frac{\sigma_{G\times j}^{2}(1-h^{2})}{h^{2}}$ with the genomic heritability $h^{2}=$ 0.5 for j=1,2, in the topical rice and barley datasets (T=2). For the DST2 maize dataset (T=3), let $\mu_{1}=100,\mu_{2}=150,\;\mu_{3}=200,\;\tau_{0}=10,\;\sigma_{G\times 1}^{2}=\sigma_{G\times 2}^{2}=20,\;\sigma_{G\times 3}^{2}=10,\;20,\;30,$ and $\sigma_{Ej}^{2}=\frac{\sigma_{G\times j}^{2}(1-h^{2})}{h^{2}}$ with $h^{2}=$ 0.5 for j=1,2,3.

**Fig. 1.**
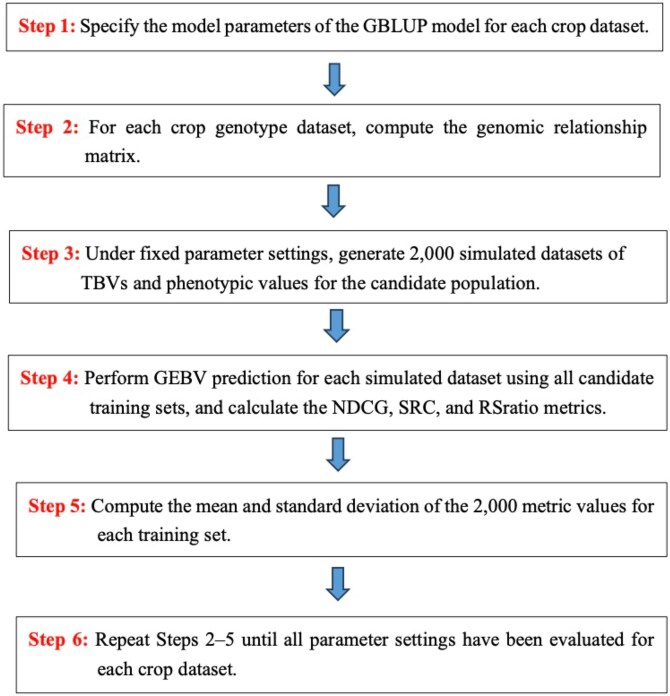
A flow diagram descripting the simulation process.

For a given setting of parameters, the genotypic values of the candidate population over the *T* environments were simulated from the corresponding multivariate normal distribution, i.e. $\;h_{cp}∼\;\mathrm{MV}N(0,\Omega_{G}⊗K_{cp}),$ and the environmental residuals $e_{cp}\;∼\;\mathrm{MV}N(0,\Omega_{E}⊗I_{cp}).$ The simulated TBVs were obtained as the sum of the fixed environmental means $\mu_{j}$ and the simulated $h_{cp},$ and the simulated phenotypic values were the simulated TBVs plus the simulated environmental residuals. Accordingly, 2,000 simulated datasets were generated for the setting. The restricted maximum likelihood estimation method in the R package sommer (Covarrubias-Pazaran 2016) was used to perform the required GEBV prediction. The means and standard deviations of the resulting NDCG@k,SRC@k, and $\;RS_{ratio}@k$ values over the 2,000 simulated datasets were calculated to compare the training sets generated from the construction methods. A flowchart of the simulation process is shown in Fig. 1, and the R scripts used to implement the simulation study are provided online.

## Results

The means and standard deviations of evaluation metrics across 2,000 simulation runs are displayed in Figs. 2–4, 5–7, and 8–10 for the tropical rice, barley, and DST2 maize datasets, respectively.

**Fig. 2.**
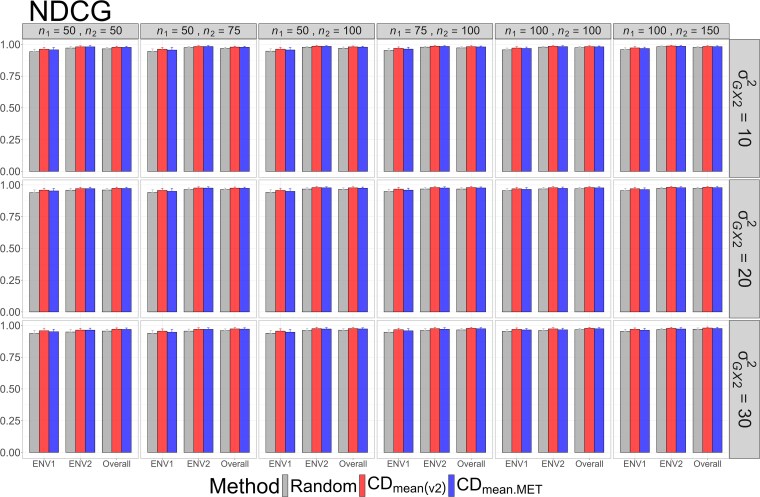
The means and standard deviations (represented by the error bars) of the NDCG values over 2,000 simulated datasets based on the 3 training set construction methods in the tropical rice dataset. Note that the overall evaluation indicates the situation of identifying the top genotypes with the highest average TBVs over environments.

**Fig. 3.**
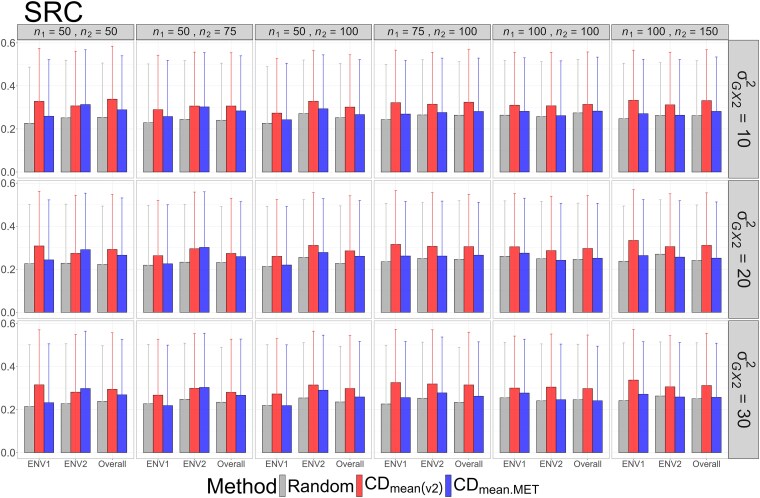
The means and standard deviations (represented by the error bars) of SRC values over 2,000 simulated datasets based on the 3 training set construction methods in the tropical rice dataset. Note that the overall evaluation indicates the situation of identifying the top genotypes with the highest average TBVs over environments.

**Fig. 4.**
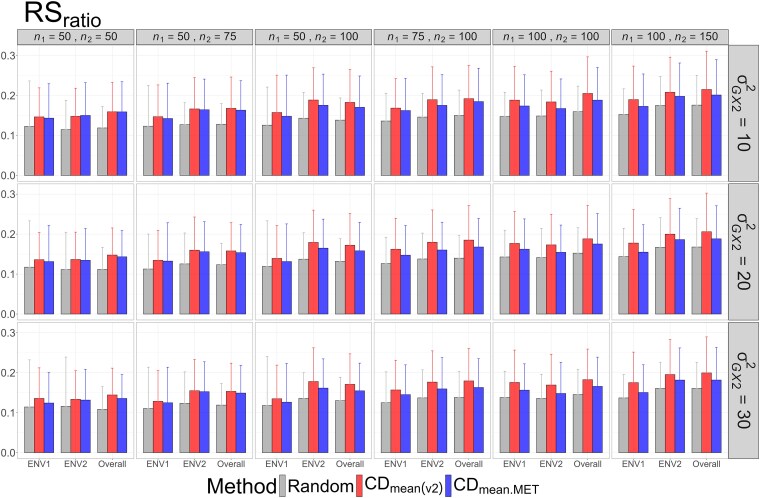
The means and standard deviations (represented by the error bars) of the RS_ratio_ values over 2,000 simulated datasets based on the 3 training set construction methods in the tropical rice dataset. Note that the overall evaluation indicates the situation of identifying the top genotypes with the highest average TBVs over environments.

**Fig. 5.**
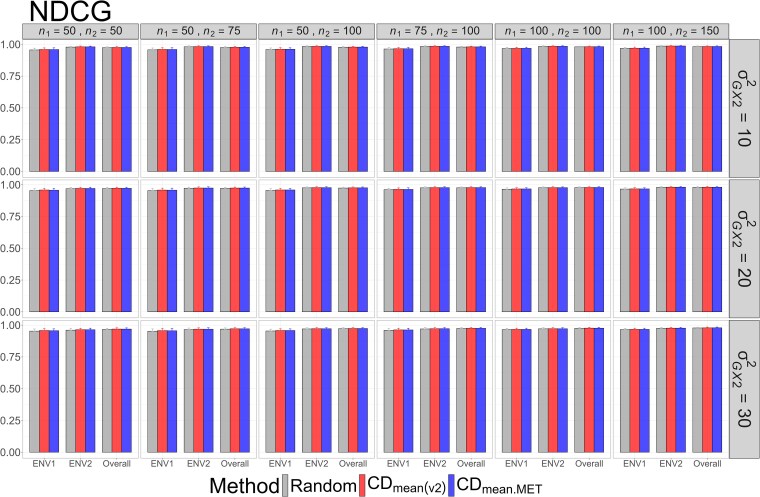
The means and standard deviations (represented by the error bars) of the NDCG values over 2,000 simulated datasets based on the 3 training set construction methods in the barley dataset. Note that the overall evaluation indicates the situation of identifying the top genotypes with the highest average TBVs over environments.

**Fig. 6.**
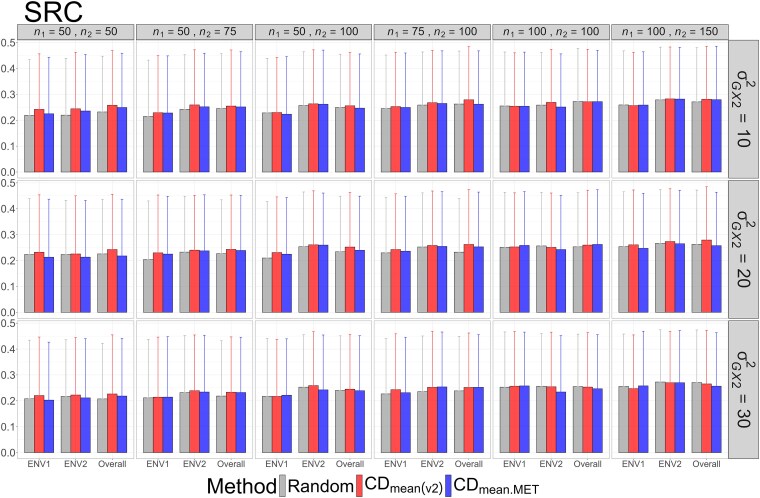
The means and standard deviations (represented by the error bars) of SRC values over 2,000 simulated datasets based on the 3 training set construction methods in the barley dataset. Note that the overall evaluation indicates the situation of identifying the top genotypes with the highest average TBVs over environments.

**Fig. 7.**
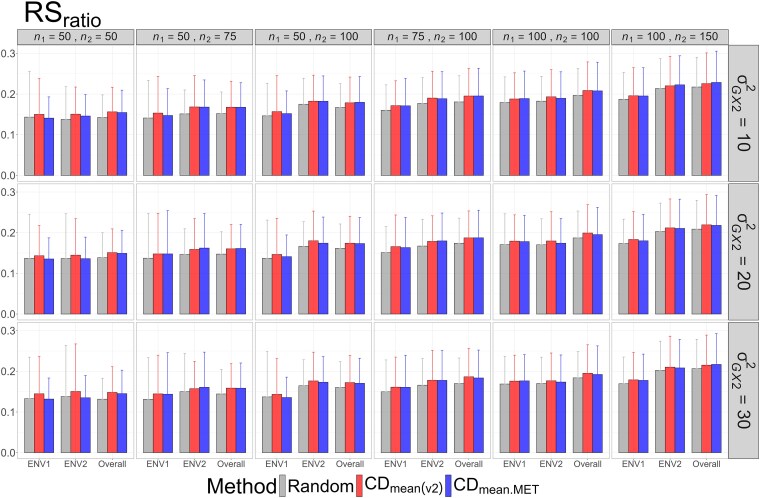
The means and standard deviations (represented by the error bars) of the RS_ratio_ values over 2,000 simulated datasets based on the 3 training set construction methods in the barley dataset. Note that the overall evaluation indicates the situation of identifying the top genotypes with the highest average TBVs over environments.

**Fig. 8.**
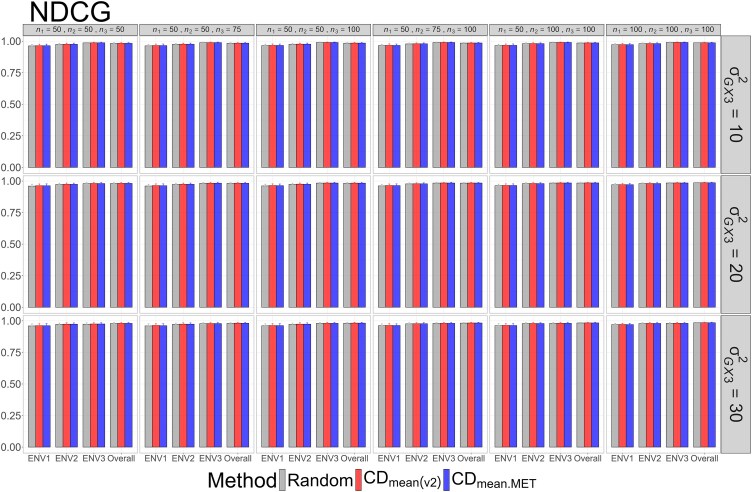
The means and standard deviations (represented by the error bars) of the NDCG values over 2,000 simulated datasets based on the 3 training set construction methods in the DST2 maize dataset. Note that the overall evaluation indicates the situation of identifying the top genotypes with the highest average TBVs over environments.

**Fig. 9.**
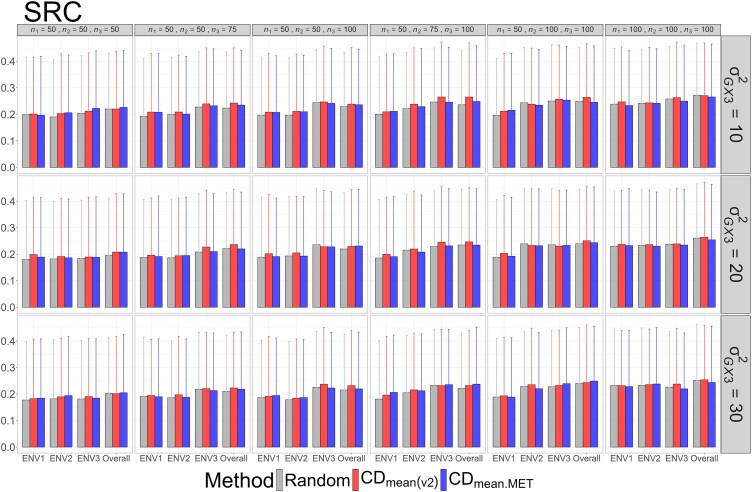
The means and standard deviations (represented by the error bars) of SRC values over 2,000 simulated datasets based on the 3 training set construction methods in the DST2 maize dataset. Note that the overall evaluation indicates the situation of identifying the top genotypes with the highest average TBVs over environments.

**Fig. 10.**
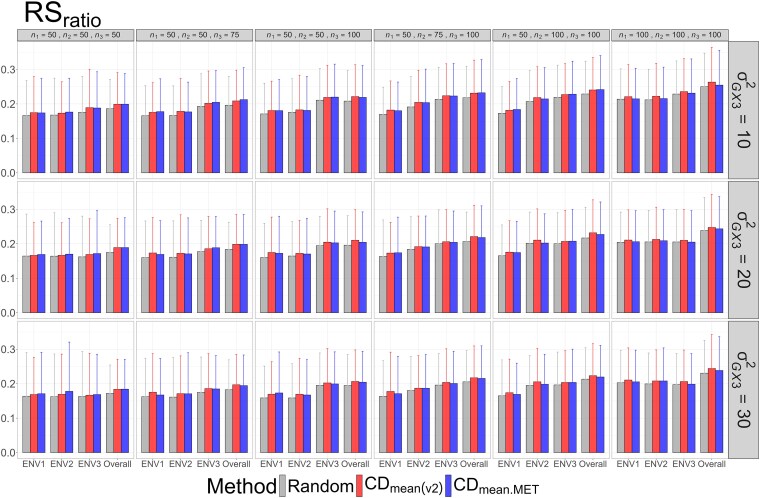
The means and standard deviations (represented by the error bars) of the RS_ratio_ values over 2,000 simulated datasets based on the 3 training set construction methods in the DST2 maize dataset. Note that the overall evaluation indicates the situation of identifying the top genotypes with the highest average TBVs over environments.

The main findings can be summarized as follows:

1. Overall performance: $CD_{\mathrm{mean}(v2)}$ generally performed at least as well as $CD_{\mathrm{mean}.\mathrm{MET}}$ and random sampling across all datasets.
2. Dataset-specific differences: In the tropical rice dataset, $CD_{\mathrm{mean}(v2)}$ substantially outperformed the other 2 methods across all evaluation metrics (Figs. 2–4). In contrast, the 3 methods exhibited more comparable performance in the barley and DST2 maize datasets (Figs. 5–10).
3. Effect of genetic variance: The performance of all construction methods declined as genetic variance increased ($\sigma_{G\times 2}^{2}$ in the tropical rice and barley datasets; $\sigma_{G\times 3}^{2}$ in the DST2 maize dataset). However, the decline is not very pronounced across training set sizes, evaluation metrics, and datasets.
4. Environment-level consistency: The relative performance of the methods in overall evaluations, i.e. identifying superior genotypes with the highest mean TBVs, aligned with their performance within individual environments.
5. Training set size: For a fixed genetic variance, increasing training set size consistently enhanced performance across all construction methods. The result also highlighted that training set size is a key factor affecting the performance.
6. Evaluation metrics: Differences among the construction methods were smaller for NDCG@k than for the other 2 metrics, SRC@k and $\;RS_{ratio}@k$.

## Discussion

A consistent observation across the results was that differences among the 3 construction methods were generally smaller for *NDCG@k* than for *SRC@k* and RSratio@k. This can be attributed to the fact that *NDCG@k* in Eq. (9) incorporates both the simulated TBV and a discount function. Because the simulated TBVs were defined as the sum of the environment-specific means and the simulated genotypic values—and the environment-specific means were set substantially larger than the genotypic values in our simulations—the metric became less sensitive to differences in DCG values between the predicted and ideal rankings. This interpretation is further supported by the observation that the estimated *NDCG@k* values exhibited much smaller standard deviations than those of *SRC@k* and RSratio@k.

Several factors influence selection efficiency in METs, including genomic relationships among genotypes (which vary across datasets), training set optimization methods, training set size, and the genetic variance and covariance of additive G × E interaction effects. In our simulations, genomic heritability ($h^{2}$) was fixed at 0.5 to reduce computational scale, though $h^{2}$ is known to strongly affect selection efficiency. To illustrate this, scenarios with $\tau_{0}=10,\;\sigma_{G\times 1}^{2}=20,\;\sigma_{G\times 2}^{2}=20,$ and $h^{2}=0.2,\;0.5,$ and 0.8 were rerun for the tropical rice dataset using random sampling. Results in Supplementary Table 1 show that all evaluation metrics increased with higher heritability, highlighting its effect on selection efficiency. In general, a simulation framework based on a limited number of scenarios such as our study may still lack some degree of practical and biological realism. Alternatively, empirical estimates of model parameters from real phenotype data could be employed to enhance biological validity.

**Fig. 11.**
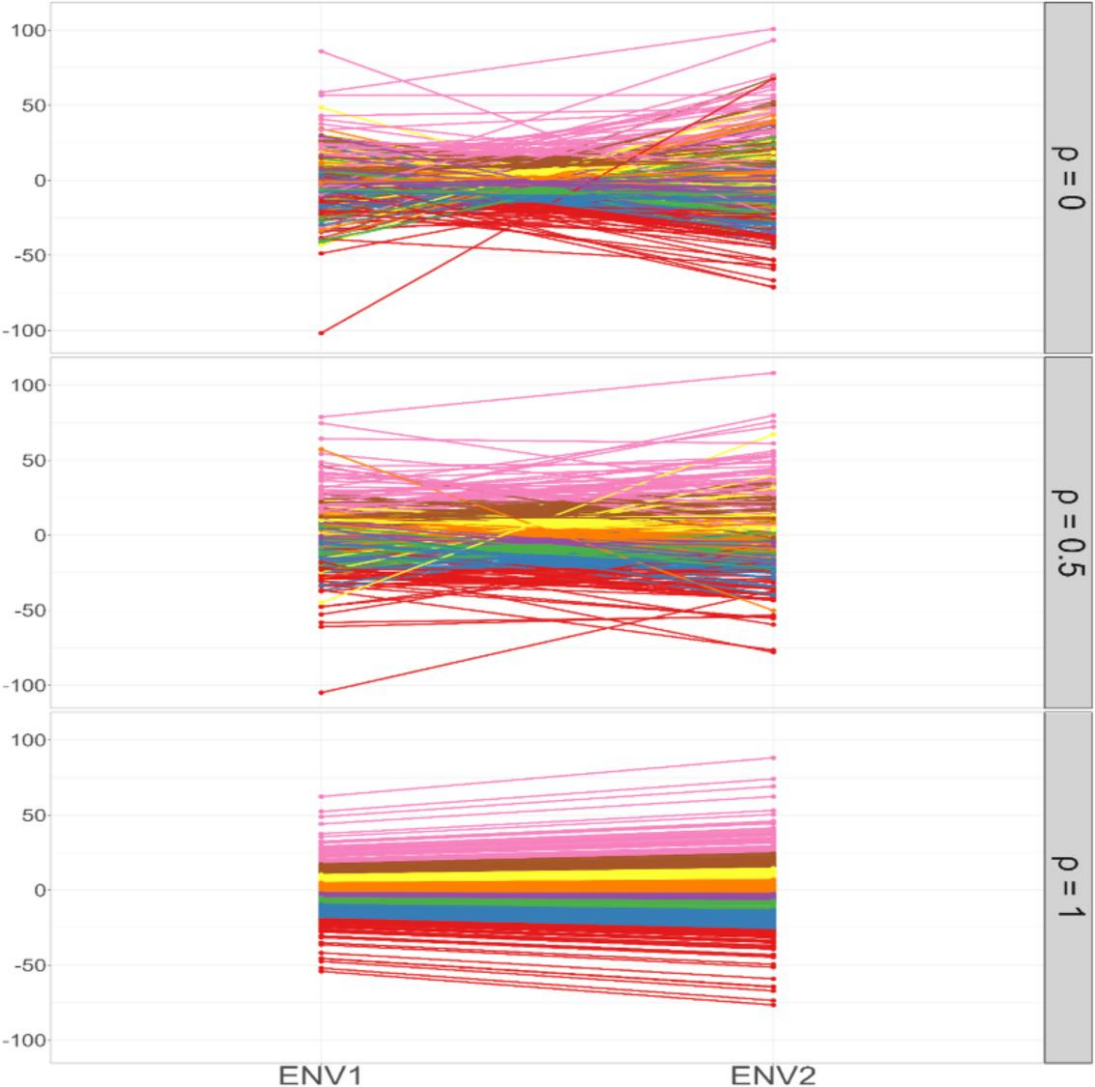
The average genotypic values of genotypes across environments at various values of the genetic correlation *ρ*, corresponding to different levels of the G × E interaction, based on 30 simulated datasets in the tropical rice dataset.

For scenarios in the tropical rice and barley datasets (T = 2), genetic covariance and variances were set as $\tau_{0}=10,\;\sigma_{G\times 1}^{2}=20,$ and $\sigma_{G\times 2}^{2}=10,\;20,\;30,$ yielding genetic correlations between environments $\rho =\frac{\tau_{0}}{\sqrt{\sigma_{G\times 1}^{2}\sigma_{G\times 2}^{2}}}=0.707,\;0.5,\;0.408$. The above main findings described for the effect of genetic variance can be restated as the performance of all construction methods improved as *ρ* increased. Furthermore, the level of G × E interactions can thus be quantified using genetic correlation. In an additional simulation based on the tropical rice dataset ($\sigma_{G\times 1}^{2}=20,\;\sigma_{G\times 2}^{2}=10,$ and ρ=0,0.5,1), average genotypic values across 30 replicates per genotype showed that as *ρ* increased, genotypic performance became more stable (Fig. 11). When ρ=1, genotypes performed consistently across environments, indicating no G × E interaction. Practically, weak G × E interactions (high *ρ*) allow breeders to select genotypes with consistently superior performance, whereas strong G × E interactions (low *ρ*) favor environment-specific genotypes.

**Fig. 12.**
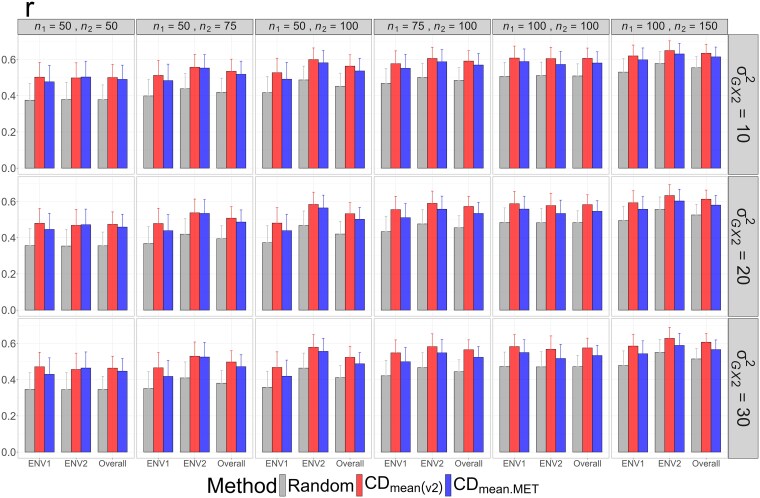
The means and standard deviations (represented by the error bars) of Pearson's correlation coefficients between the true and estimated genotypic values over 2,000 simulated datasets based on the 3 training set construction methods in the tropical rice dataset. Note that the overall evaluation indicates the situation of identifying the top genotypes with the highest average TBVs over environments.

The $CD_{\mathrm{mean}(v2)}$ method is theoretically designed to maximize the mean squared correlation between true and estimated genotypic values across all candidate genotypes and environments; however, it does not directly guarantee optimization of ranking-based metrics for identifying the top *k* genotypes. To assess the consistency between correlation-based and ranking-based metrics, Pearson's correlations between true and estimated genotypic values were examined for the tropical rice dataset (Fig. 12). The results showed that $CD_{\mathrm{mean}(v2)}$ consistently outperformed $CD_{\mathrm{mean}.\mathrm{MET}}$ and random sampling, supporting its effectiveness for selecting top-performing genotypes in METs based on ranking-oriented metrics.

In implementing $CD_{\mathrm{mean}(v2)}$, variance components $\sigma_{G\times j}^{2}$ and $\sigma_{Ej}^{2}$ were fixed at 1, and covariance $\tau_{0}$ at 0.5 in Eq. (5). To assess sensitivity, 20 training sets ($n_{1}=n_{2}=50)$ were randomly selected from the tropical rice dataset and evaluated under 5 alternative parameter settings (Table 2), and their resulting $CD_{\mathrm{mean}(v2)}$ values are displayed in Supplementary Table 2. Pearson's correlation and SRC between the resulting $CD_{\mathrm{mean}(v2)}$ values at the default setting and the other settings are shown in Table 3. Both correlation coefficients decrease as the discrepancy of the variance–covariance components increases. However, the correlation analyses across different weight settings of the variance–covariance components still showed strong concordance with the default setting, supporting its practical use. Nevertheless, robustness verification based solely on these limited rank-concordance tests may be insufficient. A more comprehensive sensitivity analysis—incorporating a broader range of parameter values, runtime comparisons, and variations in evaluation metrics—would provide stronger and more convincing evidence.

**Table 2.** Setting parameters in calculating $CD_{\mathrm{mean}(v2)}$ values for 20 random training sets chosen from the tropical rice dataset.

	Set0	Set1	Set2	Set3	Set4	Set5
$\sigma_{G\times 1}^{2}$	1	1	1	1	1	1
$\sigma_{G\times 2}^{2}$	1	1	1	2	5	1
$\tau_{0}$	0.5	0.2	0.8	0.5	0.5	0.5
$\sigma_{E1}^{2}$	1	1	1	1	1	1
$\sigma_{E2}^{2}$	1	1	1	1	2	5

**Table 3.** Pearson's correlations (r) and SRC over the CD_mean(v2)_ values of 20 random samples between the default setting (Set0) and the other 5 settings in the tropical rice dataset.

	Set1	Set2	Set3	Set4	Set5
r	0.9952	0.9939	0.9944	0.9908	0.9691
SRC	0.9880	0.9835	0.9880	0.9835	0.9429

The optimization algorithm implemented in TrainSel combines a GA with a simulated annealing algorithm (SAA), in which SAA steps are executed within each GA iteration to reduce the risk of convergence to local optima. TrainSel further offers 2 optimization settings, referred to as low-complexity and high-complexity modes. We first compared the performance of training sets generated by our approach with those obtained using the $CD_{\mathrm{mean}}$ criterion under the low-complexity setting in TrainSel, based on the tropical rice dataset. The low-complexity setting relies solely on a GA, making it comparable to the metaheuristic optimization strategy employed in our approach. Table 4 summarizes the runtime, the number of GA iterations required to generate training sets, the average CD values across all candidate genotypes, and the means and standard deviations of NDCG, SRC, and RS_ratio_ for identifying the top 5% of genotypes with the highest mean TBVs under the simulation setting $\tau_{0}=10,\;\sigma_{G\times 1}^{2}=20,$ and $\sigma_{G\times 2}^{2}=20$. The results indicate that our approach produced training sets with higher CD values and superior ranking-based performance, albeit at a substantially higher computational cost than the TrainSel implementation. To increase the likelihood of identifying near-global optima, we enforced a minimum of 12,000 GA iterations before applying stopping rules in our metaheuristic algorithm. This design choice largely explains the increased runtime observed for our approach.

**Table 4.** Runtime and number of GA iterations, mean CD values, and the means (standard deviations) of NDCG, SRC, and RSratio across 2,000 simulations for identifying the top 5% of genotypes with the highest mean TBVs under $\tau_{0}=10,\;\sigma_{G\times 1}^{2}=20,$ and $\sigma_{G\times 2}^{2}=20$, based on the tropical rice dataset.

	Approach	n_1_ = 50, n_2_ = 50	n_1_ = 50, n_2_ = 75	n_1_ = 50, n_2_ = 100	n_1_ = 75, n_2_ = 100	n_1_ = 100, n_2_ = 100	n_1_ = 100, n_2_ = 150
Time (hr)	Ours	7.0803	9.9047	11.2750	11.2603	17.4267	24.2519
	TrainSel	2.3751	2.3357	6.1228	7.6014	11.9916	15.4421
Iterations	Ours	12001	12117	12001	12350	12001	12313
	TrainSel	994	1189	1656	1555	2660	1656
Mean CD	Ours	0.1920	0.2212	0.2478	0.2748	0.2993	0.3419
	TrainSel	0.1390	0.1584	0.1753	0.1955	0.2132	0.2474
NDCG	Ours	0.9730 (0.0089)	0.9748 (0.0086)	0.9766 (0.0083)	0.9791 (0.0074)	0.9792 (0.0074)	0.9811 (0.0069)
	TrainSel	0.9667 (0.0115)	0.9718 (0.0097)	0.9740 (0.0089)	0.9752 (0.0084)	0.9754 (0.0082)	0.9763 (0.0077)
SRC	Ours	0.2921 (0.2555)	0.2734 (0.2564)	0.2853 (0.2559)	0.3051 (0.2428)	0.2963 (0.2458)	0.3110 (0.2438)
	TrainSel	0.2393 (0.2718)	0.2538 (0.2618)	0.2469 (0.2626)	0.2482 (0.2558)	0.2398 (0.2558)	0.2381 (0.2500)
RS_ratio_	Ours	0.1476 (0.0677)	0.1579 (0.0709)	0.1721 (0.0796)	0.1851 (0.0861)	0.1881 (0.0834)	0.2058 (0.0967)
	TrainSel	0.1304 (0.0607)	0.1452 (0.0635)	0.1581 (0.0667)	0.1660 (0.0714)	0.1686 (0.0728)	0.1751 (0.0752)

Results are shown for our approach and $CD_{\mathrm{mean}}$ with the low-complexity setting in TrainSel. Note that the programs were run by a PC with 2.50-GHz, 8-core Intel Core i9-11900.

Furthermore, we implemented $CD_{\mathrm{mean}(v2)}$ within the TrainSel framework using both the low-complexity and high-complexity settings and compared its performance with that of $CD_{\mathrm{mean}}$. For the high-complexity setting, 10 SAA iterations were performed within each GA iteration. The resulting runtimes and average CD values are summarized in Table 5. These results show that $CD_{\mathrm{mean}(v2)}$ under the low-complexity setting produced training sets with CD values comparable to those obtained under the high-complexity setting and consistently higher than those produced by $CD_{\mathrm{mean}}$ under either setting. Compared with the results in Table 4, $CD_{\mathrm{mean}(v2)}$ with the low complexity yielded training sets with slightly higher CD values than those generated by our original approach. Although $CD_{\mathrm{mean}(v2)}$ and $CD_{\mathrm{mean}}$ are theoretically equivalent, their implementations differ computationally. In TrainSel, $CD_{\mathrm{mean}}$ is computed using a matrix inversion of dimension $T\times n_{c}$ (see Eq. (2) in Rio et al. (2022) and the formulation in Akdemir et al. (2021)), whereas $CD_{\mathrm{mean}(v2)}$ involves a matrix inversion of dimension $n_{tr}$. Because the total training set size $n_{tr}$ (the sum of training set sizes across *T* environments) is typically much smaller than $T\times n_{c}$, these differences in computational formulation may lead to differences in optimization behavior and resulting training sets. Consequently, we recommend combining $CD_{\mathrm{mean}(v2)}$with the low-complexity setting for practical breeding programs.

**Table 5.** Runtime and mean CD values implementing $CD_{\mathrm{mean}(v2)}$ and $CD_{\mathrm{mean}}$ under the low- and high-complexity settings in TrainSel, based on the tropical rice dataset.

Method	Setting		n_1_ = 50, n_2_ = 50	n_1_ = 50, n_2_ = 75	n_1_ = 50, n_2_ = 100	n_1_ = 75, n_2_ = 100	n_1_ = 100, n_2_ = 100	n_1_ = 100, n_2_ = 150
CD_mean_	Low-complexity	Time (hr)	2.3751	2.3357	6.1228	7.6014	11.9916	15.4421
		Mean CD	0.1390	0.1584	0.1753	0.1955	0.2132	0.2474
	High-complexity	Time (hr)	3.7626	5.5672	7.8593	11.5196	13.4867	20.5287
		Mean CD	0.1389	0.1581	0.1758	0.1956	0.2127	0.2474
CD_mean(v2)_	Low-complexity	Time (hr)	2.2077	2.3909	4.0095	6.3728	8.8422	9.8657
		Mean CD	0.2020	0.2333	0.2606	0.2890	0.3139	0.3574
	High-complexity	Time (hr)	3.0681	4.9006	7.4577	8.6765	11.1237	17.0755
		Mean CD	0.2021	0.2334	0.2611	0.2886	0.3139	0.3578

Note that the programs were run by a PC with 2.50-GHz, 8-core Intel Core i9-11900.

Several limitations of this study warrant consideration and suggest directions for future research. First, the simulation experiments were conducted under an additive MGE model with a common covariance, whereas real breeding data may exhibit more complex genetic architectures, including dominance and epistasis effects, as well as heterogeneous covariance structures across environments. Extending the proposed framework to accommodate these additional sources of genetic variation would be a natural next step. Second, our optimization procedures focused on untargeted testing set selection and did not incorporate prior information from specific target populations or environments. Future work could explore hybrid strategies that integrate CD-based criteria with targeted or adaptive optimization approaches. Finally, while the low-complexity implementation of $CD_{\mathrm{mean}(v2)}$ offers a favorable balance between performance and computational cost, further algorithmic improvements may enhance scalability for very large candidate populations and high-dimensional MET designs.

## Supplementary Material

jkag031_Supplementary_Data

## Data Availability

All phenotype and genotype datasets analyzed in this study are publicly available and can be downloaded from Figshare at https://doi.org/10.6084/m9.figshare.29453807.v1. In addition, 4 R scripts are available in a GitHub repository (https://github.com/simonb08601003/OTSGSISGME/tree/main): (1) Gen-Opt-TRS, which implements the $CD_{\mathrm{mean}(v2)}$ method using the proposed GA-based metaheuristic algorithm; (2) Gen-Simu-Exp, which generates the simulated datasets; (3) Cal-Rank-Mer, which computes the NDCG, SRC, and RS_ratio_ metrics; and (4) CDmean(v2)-TrainSel, which implements $CD_{\mathrm{mean}(v2)}$ and $CD_{\mathrm{mean}(v2)}$ under the low- and high-complexity settings in TrainSel. Supplemental material available at *G3* online.
