# Supplementary material for: Optimizing training sets for genomic selection to identify superior genotypes across multiple environments
Source: G3 (Bethesda). 2026 Feb 10;16(4):jkag031. doi: 10.1093/g3journal/jkag031 (PMC13042293; doi:10.1093/g3journal/jkag031)
Supplement: jkag031_Supplementary_Data [file jkag031_supplementary_data.zip › Table_S1_G3-2026-406533.docx]

Table S1. The mean and standard deviation (in parentheses) of evaluation metrics in the simulation study with various heritability values based on the random sampling method.

| NDCG | | | | | | | | | |
| --- | --- | --- | --- | --- | --- | --- | --- | --- | --- |
|  |  | n_1_ = 50, n_2_ = 50 | n_1_ = 50, n_2_ = 100 | | n_1_ = 100, n_2_ = 100 | | n_1_ = 100, n_2_ = 150 | |  |
| h^2^ = 0.2 | ENV1 | 0.9373 (0.0220) | 0.9407 (0.0212) | | 0.9497 (0.0197) | | 0.9514 (0.0183) | |  |
|  | ENV2 | 0.9368 (0.0203) | 0.9474 (0.0193) | | 0.9488 (0.0174) | | 0.9555 (0.0159) | |  |
|  | Overall | 0.9482 (0.0162) | 0.9551 (0.0150) | | 0.9588 (0.0131) | | 0.9631 (0.0120) | |  |
| h^2^ = 0.5 | ENV1 | 0.9528 (0.0171) | 0.9550 (0.0161) | | 0.9653 (0.0123) | | 0.9665 (0.0116) | |  |
|  | ENV2 | 0.9517 (0.0161) | 0.9635 (0.0123) | | 0.9641 (0.0124) | | 0.9712 (0.0102) | |  |
|  | Overall | 0.9608 (0.0125) | 0.9683 (0.0104) | | 0.9714 (0.0097) | | 0.9757 (0.0086) | |  |
| h^2^ = 0.8 | ENV1 | 0.9620 (0.0131) | 0.9641 (0.0120) | | 0.9740 (0.0100) | | 0.9750 (0.0100) | |  |
|  | ENV2 | 0.9602 (0.0136) | 0.9724 (0.0105) | | 0.9726 (0.0107) | | 0.9802 (0.0086) | |  |
|  | Overall | 0.9678 (0.0109) | 0.9755 (0.0093) | | 0.9784 (0.0086) | | 0.9828 (0.0072) | |  |
|  |  |  | |  | |  | |  | |
| SRC | | | | | | | | | |
|  |  | n_1_ = 50, n_2_ = 50 | n_1_ = 50, n_2_ = 100 | | n_1_ = 100, n_2_ = 100 | | n_1_ = 100, n_2_ = 150 | |  |
| h^2^ = 0.2 | ENV1 | 0.1347 (0.2978) | 0.1408 (0.2904) | | 0.1831 (0.2832) | | 0.1818 (0.2763) | |  |
|  | ENV2 | 0.1559 (0.2979) | 0.1833 (0.2794) | | 0.1811 (0.2851) | | 0.2055 (0.2729) | |  |
|  | Overall | 0.1556 (0.2857) | 0.1826 (0.2739) | | 0.1928 (0.2755) | | 0.2026 (0.2599) | |  |
| h^2^ = 0.5 | ENV1 | 0.2701 (0.2664) | 0.2473 (0.2681) | | 0.2904 (0.2530) | | 0.2840 (0.2511) | |  |
|  | ENV2 | 0.2606 (0.2648) | 0.2950 (0.2469) | | 0.2891 (0.2528) | | 0.3149 (0.2388) | |  |
|  | Overall | 0.2634 (0.2596) | 0.2802 (0.2536) | | 0.2834 (0.2454) | | 0.3010 (0.2428) | |  |
| h^2^ = 0.8 | ENV1 | 0.3487 (0.2417) | 0.3281 (0.2531) | | 0.3857 (0.2382) | | 0.3693 (0.2453) | |  |
|  | ENV2 | 0.3432 (0.2481) | 0.3994 (0.2279) | | 0.3792 (0.2409) | | 0.4244 (0.2299) | |  |
|  | Overall | 0.3407 (0.2369) | 0.3562 (0.2306) | | 0.3621 (0.2326) | | 0.3811 (0.2353) | |  |
|  |  |  |  | |  | |  | |  |
| RS_ratio_ | | | | | | | | | |
|  |  | n_1_ = 50, n_2_ = 50 | n_1_ = 50, n_2_ = 100 | | n_1_ = 100, n_2_ = 100 | | n_1_ = 100, n_2_ = 150 | |  |
| h^2^ = 0.2 | ENV1 | 0.1157 (0.1856) | 0.1229 (0.1886) | | 0.1115 (0.1067) | | 0.1155 (0.1170) | |  |
|  | ENV2 | 0.1630 (0.2733) | 0.1393 (0.1939) | | 0.1349 (0.1776) | | 0.1303 (0.1288) | |  |
|  | Overall | 0.0934 (0.0965) | 0.1061 (0.0934) | | 0.1093 (0.0586) | | 0.1203 (0.0590) | |  |
| h^2^ = 0.5 | ENV1 | 0.1276 (0.1142) | 0.1247 (0.0913) | | 0.1595 (0.0690) | | 0.1629 (0.0656) | |  |
|  | ENV2 | 0.1289 (0.1245) | 0.1578 (0.0796) | | 0.1584 (0.0759) | | 0.1932 (0.0770) | |  |
|  | Overall | 0.1230 (0.0615) | 0.1528 (0.0612) | | 0.1706 (0.0672) | | 0.1981 (0.0773) | |  |
| h^2^ = 0.8 | ENV1 | 0.1542 (0.0620) | 0.1594 (0.0610) | | 0.2339 (0.0857) | | 0.2404 (0.0919) | |  |
|  | ENV2 | 0.1535 (0.0739) | 0.2303 (0.0845) | | 0.2315 (0.0873) | | 0.3081 (0.1071) | |  |
|  | Overall | 0.1633 (0.0608) | 0.2212 (0.0796) | | 0.2502 (0.0920) | | 0.3024 (0.1052) | |  |
