# Supplementary material for: Optimizing training sets for genomic selection to identify superior genotypes across multiple environments
Source: G3 (Bethesda). 2026 Feb 10;16(4):jkag031. doi: 10.1093/g3journal/jkag031 (PMC13042293; doi:10.1093/g3journal/jkag031)
Supplement: jkag031_Supplementary_Data [file jkag031_supplementary_data.zip › Table_S2_G3-2026-406533.docx]

Table S2. The CD_mean(v2)_ values and rankings (in parentheses) for the 20 training sets under the parameter settings.

| Training set | Set0 | Set1 | Set2 | Set3 | Set4 | Set5 |
| --- | --- | --- | --- | --- | --- | --- |
| D1 | 0.1873 (1) | 0.1649 (1) | 0.2075 (1) | 0.199 (1) | 0.1981 (1) | 0.1346 (2) |
| D2 | 0.1859 (2) | 0.1635 (3) | 0.2063 (2) | 0.1979 (2) | 0.1970 (2) | 0.1341 (3) |
| D3 | 0.1859 (3) | 0.1638 (2) | 0.2060 (3) | 0.1967 (3) | 0.1957 (3) | 0.1366 (1) |
| D4 | 0.1824 (4) | 0.1594 (5) | 0.2034 (4) | 0.1939 (4) | 0.1927 (5) | 0.1307 (8) |
| D5 | 0.1819 (5) | 0.1595 (4) | 0.2022 (7) | 0.1938 (5) | 0.1930 (4) | 0.1317 (5) |
| D6 | 0.1816 (6) | 0.1586 (6) | 0.2028 (5) | 0.1926 (7) | 0.1913 (7) | 0.1314 (6) |
| D7 | 0.1809 (7) | 0.1575 (9) | 0.2023 (6) | 0.1929 (6) | 0.1918 (6) | 0.1284 (12) |
| D8 | 0.1809 (8) | 0.1580 (8) | 0.2020 (8) | 0.1917 (8) | 0.1904 (8) | 0.1312 (7) |
| D9 | 0.1807 (9) | 0.1585 (7) | 0.2009 (10) | 0.1910 (10) | 0.1898 (10) | 0.1319 (4) |
| D10 | 0.1803 (10) | 0.1571 (10) | 0.2016 (9) | 0.1914 (9) | 0.1901 (9) | 0.1289 (10) |
| D11 | 0.1784 (11) | 0.1551 (11) | 0.1999 (12) | 0.1893 (11) | 0.1879 (11) | 0.1296 (9) |
| D12 | 0.1781 (12) | 0.1546 (12) | 0.1999 (11) | 0.1884 (13) | 0.1867 (13) | 0.1285 (11) |
| D13 | 0.1766 (13) | 0.1527 (15) | 0.1988 (13) | 0.1885 (12) | 0.1871 (12) | 0.1250 (15) |
| D14 | 0.1764 (14) | 0.1534 (13) | 0.1977 (14) | 0.1867 (15) | 0.1851 (15) | 0.1269 (13) |
| D15 | 0.1756 (15) | 0.1528 (14) | 0.1965 (15) | 0.1873 (14) | 0.1863 (14) | 0.1256 (14) |
| D16 | 0.1719 (16) | 0.1493 (16) | 0.1926 (18) | 0.1843 (16) | 0.1835 (16) | 0.1214 (18) |
| D17 | 0.1714 (17) | 0.1483 (17) | 0.1929 (16) | 0.1819 (17) | 0.1804 (18) | 0.1231 (16) |
| D18 | 0.1709 (18) | 0.1482 (18) | 0.1919 (19) | 0.1819 (18) | 0.1805 (17) | 0.1204 (19) |
| D19 | 0.1709 (19) | 0.1477 (19) | 0.1926 (17) | 0.1814 (19) | 0.1798 (19) | 0.1230 (17) |
| D20 | 0.1647 (20) | 0.1433 (20) | 0.1845 (20) | 0.1751 (20) | 0.1739 (20) | 0.1185 (20) |
